# Mental stress elicits sustained and reproducible increases in skin sympathetic nerve activity

**DOI:** 10.1002/phy2.2

**Published:** 2013-05-07

**Authors:** Matthew D Muller, Charity L Sauder, Chester A Ray

**Affiliations:** Penn State Heart and Vascular Institute, Department of Cellular and Molecular Physiology, Clinical Research Center, Pennsylvania State University College of Medicine, The Milton S. Hershey Medical CenterHershey, PA, 17033

**Keywords:** Blood pressure, heart rate, psychosocial stress, reproducibility of results

## Abstract

Mental stress (MS) is a known trigger of myocardial infarction and sudden death. By activating the sympathetic nervous system, MS may have deleterious effect on the cardiovascular system but this process is not completely understood. The primary aim of this study was to quantify the effect of MS on skin sympathetic nerve activity (SSNA). The secondary aim was to determine the reproducibility of SSNA to MS within a given day and ∼1 week later. Ten subjects (26 ± 1 year) performed two bouts of mental arithmetic lasting 3 min. The bouts were separated by 45 min. One week later the subjects returned to repeat MS. All experiments were conducted in the supine posture during the morning hours. To maintain neutral skin temperature, each subject wore a custom suit (34–35°C). Skin blood flow and sweat rate were measured on the dorsal foot. MS elicited a marked increase in SSNA within the first 10 sec (184 ± 42%; *P* < 0.01) in all subjects, which was less during the remaining period of MS, but remained elevated (87 ± 20; *P* < 0.01). The pattern of responses to MS was unchanged during the second bout (10 sec, 247 ± 55%; 3 min average, 133 ± 29%) and during the retest 1 week later (10 sec, 196 ± 55%; 3 min average, 117 ± 36%). MS did not significantly affect cutaneous vascular conductance or sweat rate during any trial. In summary, MS elicits robust and reproducible increases in SSNA in humans, which may be followed over time to observe alterations in the regulation of the autonomic nervous system.

## Introduction

Psychological stress is an established trigger for adverse cardiovascular events (Matthews et al. 2004; Dimsdale 2008), but the underlying mechanisms are not entirely clear. Stimuli that elicit psychological stress include public speaking, fast-paced mathematical calculations, argument with coworkers, and the panic associated with natural disaster such as earthquakes and floods (Dimsdale 2008). As these stimuli are not uncommon, a better understanding of how the body responds to psychological stress may allow for therapies to improve clinical outcomes.

Recent laboratory experiments (Carter and Ray 2009; Durocher et al. 2009, 2011a,b; Klein et al. 2010; Ray and Carter 2010; Schwartz et al. 2011; Carter et al. 2012; Yang et al. 2012) have indicated that the sympathetic nervous system plays a pivotal role in the acute hemodynamic adjustments to mental stress (MS) (i.e., fast-paced verbal mental arithmetic). Heart rate (HR) and mean arterial pressure (MAP) consistently increase during 3- to 5-min bouts of MS whereas muscle sympathetic nerve activity (MSNA) responses are variable between subjects (Carter and Ray 2009). Skin sympathetic nerve activity (SSNA), reflective of sympathetic outflow to the cutaneous vasculature, has not been systematically studied in response to MS. Early experiments demonstrated that SSNA responds to arousal stimuli (e.g., sudden touch, loud noise, electrical stimulation, stressful conversation) (Delius et al. 1972; Hagbarth et al. 1972; Normell and Wallin 1974) and also participates in reflex control of skin blood flow and sweating (Bini et al. 1980; Oberle et al. 1988). Prior studies have suggested that increases in SSNA to an arousal stimulus may be attenuated over time (i.e., habituation following an initial “novelty effect”) (Delius et al. 1972; Hagbarth et al. 1972; Oberle et al. 1988), but quantitative data regarding SSNA responses to MS are scarce (Iwase et al. 1997; Yamamoto et al. 1997). Quantifying SSNA responses to mental arithmetic (i.e., a stimulus that engages cognitive function continuously) may allow for clinical assessment of interventions that might modify the autonomic nervous system.

The purpose of this study was to quantify SSNA responses to a standardized 3-min bout of mental arithmetic and to determine the reproducibility within and between days. We hypothesized that the SSNA response to fast-paced verbal mental arithmetic would be greatest within the first 10 sec of stimulus onset (arousal response), responses would be sustained throughout, and that the total SSNA response would be comparable between trials.

## Methods

### Subjects

Ten subjects (six men, four women) with a mean age of 26 ± 1 year, height of 1.76 ± 0.04 m, and weight of 76.4 ± 4.2 kg participated in this study. Subjects were determined to be healthy via medical history and physical examination and were not taking medication. All subjects refrained from caffeine, alcohol, and exercise for 24 h before the study and arrived to the laboratory in a semi-fasted state (i.e., at least 4–6 h after their last meal). The study protocols were approved in advance by the Institutional Review Board of the Penn State Milton S. Hershey Medical Center and conformed to the Declaration of Helsinki. Each participant provided written informed consent.

### Protocol

This study employed a repeated measures design. All experiments were conducted in the supine posture during the morning hours (8:00 and 11:30 am) in a dimly lit, quiet, thermoneutral laboratory (22–25°C). To maintain neutral skin temperature, each subject wore a custom-designed tube-lined suit (Med-Eng Systems, Ottawa, Ontario, Canada). The suit covered the entire body except for the head, hands, feet, and the left lower leg (i.e., where SSNA was measured). Water was perfused at 34–35°C for the entire study.

Upon arrival at the laboratory, subjects were outfitted with several hemodynamic and thermal monitoring devices (see below). They were familiarized to the procedures and encouraged to respond as quickly and correctly as possible during the upcoming MS trial. Following successful nerve recording and attachment of measuring devices, resting blood pressure was obtained. Next, a 5-min baseline occurred to quantify resting SSNA. The total duration from when the subject was placed in the thermoneutral suit until the start of baseline was always >30 min. The baseline period was followed by 3 min of fast paced verbal mental arithmetic, as previously described for our laboratory (Carter et al. 2002, 2005a,b; Kuipers et al. 2008; Ray and Carter 2010). Specifically, subjects were provided a two or three digit number and were instructed to subtract the number seven as fast as possible. The eyes remained closed during testing and subjects were encouraged to speak softly but quickly. Investigators provided a new number from which to subtract every 5–10 sec. A 3-min recovery period occurred following MS. These procedures were repeated ∼45 min later (Trial 2). On a separate day (∼7–10 days later), subjects (*n* = 9) returned to the laboratory and underwent the same procedures (Trial 3).

### Instrumentation and measurements

Mean skin temperature was measured via weighted average of three thermocouples (model TC-1000, Sable Systems, Las Vegas, NV) attached to the skin (Burton 1935; Sawka et al. 1988). These thermocouples were underneath the suit, but insulated from contacting the suit itself. Tympanic temperature, an index of core temperature (Brinnel and Cabanac 1988), was measured before and after testing with an automated device (Genius 3, AccuSystem, Mansfield, MA). HR was measured via 3-lead electrocardiogram and beat-by-beat MAP was determined by photoplethysmography (Finometer, FMS, The Netherlands). Resting blood pressures were obtained by automated oscillometry (Dinamap XL, Critikon/GE, Tampa, FL).

Multifiber recordings of SSNA were made with a tungsten microelectrode inserted in the peroneal nerve at the fibular head (always the left leg). A reference electrode was placed 2–3 cm from the recording electrode. The recording electrode was adjusted until a site was found in which SSNA bursts were clearly identified using previously established criteria (Delius et al. 1972; Vallbo et al. 1979). In brief, these included the following: (1) light stroking of the skin within the innervated region resulted in afferent discharge and (2) deep inspiration and arousal stimuli resulted in a nonpulse-synchronous activity. The nerve signal was amplified, passed through a band-pass filter with a bandwidth of 700–2000 Hz and integrated with a time constant of 0.1 sec (Iowa Bioengineering, Iowa City, IA). Mean voltage neurograms were visually displayed and recorded on a data-acquisition system (16SP Power Lab, AD Instruments, New Castle, Australia) and routed to a loudspeaker for monitoring throughout the study. SSNA responsiveness to auditory stimuli and deep inspiration was confirmed at the very end of experiments to ensure a consistent recording site.

Once the recording nerve site was established, two skin blood flow lasers (Moor Instruments, Wilmington, DE, local heater set at 34°C) and one thermocouple were carefully attached to the dorsal foot (within the region of innervation on the left foot) (Sugenoya et al. 1990; Sawasaki et al. 2001; Wilson et al. 2004). Sweat rate was measured on the contralateral dorsal foot via capacitance hygrometry (Vaisala, Woburn, MA) by perfusing 100% nitrogen at a flow rate of 150 mL/min through a ventilated capsule (surface area = 2.0 cm^2^). Perception of stress (0 = not stressful, 1 = somewhat stressful, 2 = stressful, 3 = very stressful, and 4 = very very stressful) (Callister et al. 1992) was quantified after the bout of mental arithmetic. Thermal sensation and thermal comfort (DuBois et al. 1990) were also determined before and after testing.

For all trials, beat-by-beat physiological measurements were recorded electronically and analyzed offline (16SP, Powerlab, ADInstruments, New Castle, Australia). Perceptual variables were obtained by verbal report.

### Data analysis and statistics

Sympathetic recordings that demonstrated possible electrode site shifts or electromyographic artifact were excluded from analysis (*n* = 1, leaving nine full data sets for Trials 1–3). Consistent with previous experiments in our laboratory (Ray and Wilson 2004; Wilson et al. 2004, 2006) and others (Vissing et al. 1991; Iwase et al. 1997; Toma et al. 2011), SSNA was expressed as a percent change in total area under the mean voltage neurogram relative to the preceding baseline (5 min average). This was achieved using computer software (Chart 5, ADInstruments) and the focus was on the first 10 sec of MS, as well as averages of minutes 1–3. Repeated measures analysis of variance (ANOVA) (three trials × four time points) was used to determine whether the pattern of SSNA responsiveness was different between trials. Cutaneous vascular conductance (CVC) was calculated as the quotient of skin blood flow flux and MAP. Changes in CVC due to MS were expressed as a percent change from the preceding 5-min baseline. Absolute changes in HR, MAP, sweat rate, and skin temperature were also determined. Intraclass correlations were used to compare SSNA responses to MS between trials. All values are reported as mean ± SE and *P* values <0.05 were considered statistically significant.

## Results

Resting systolic blood pressure (115 ± 2, 116 ± 1, and 115 ± 2 mmHg), resting diastolic blood pressure (64 ± 2, 63 ± 2, and 66 ± 2 mmHg), and resting CVC (0.23 ± 0.02, 0.24 ± 0.01, 0.24 ± 0.01 au) were comparable prior to Trial 1, Trial 2, and Trial 3, respectively. Mean skin temperature was stable during Trial 1 (before: 34.6 ± 0.1°C; after: 34.6 ± 0.1°C), Trial 2 (before: 34.8 ± 0.2°C; after: 34.9 ± 0.2°C), and Trial 3 (before: 34.2 ± 0.3°C; after: 34.2 ± 0.3°C). Tympanic temperature was also not affected by the mental arithmetic trials. Before and after MS, individuals consistently reported that they felt “neutral” and “comfortable.”

**Figure 1:**
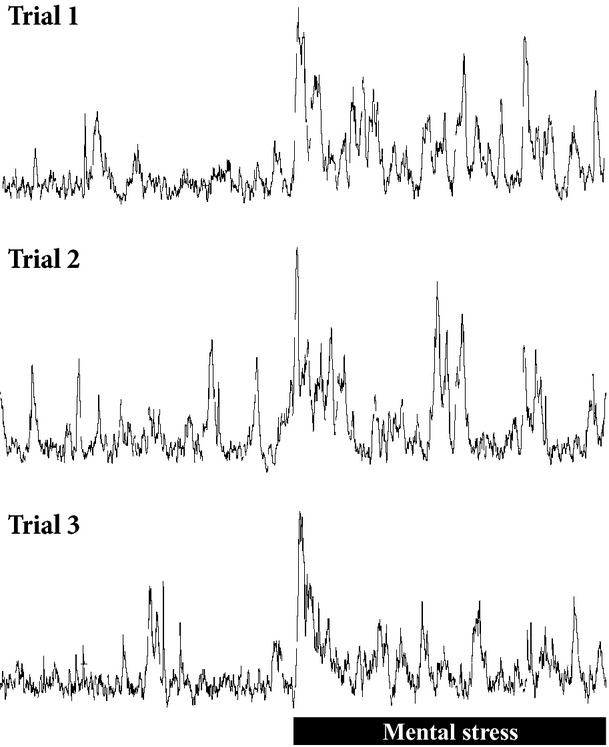
Representative recordings of skin sympathetic nerve activity (SSNA) in response to mental stress (fast-paced verbal mental arithmetic). The above figure includes the last 30 sec of baseline and the first 30 sec of mental stress. Trial 1 and 2 occurred on the same day and were separated by 45 min. Trial 3 occurred more than 1 week later.

MS significantly increased HR and MAP, but the magnitude of increase was not different between trials (Table 1). As documented in Figure 1, MS also increased SSNA relative to baseline (main effect for time <0.001), but this increase was not different between trials (main effect for trial *P* = 0.496, trial by time interaction *P* = 0.536). Furthermore, skin blood flow and sweat rate were not different between trials (Table 1). In a similar way, dorsal foot temperature was stable during Trial 1 (before: 27.6 ± 0.4°C; after: 27.7 ± 0.4°C), Trial 2 (before: 27.1 ± 0.6°C; after: 27.1 ± 0.6°C), and Trial 3 (before: 27.1 ± 0.6°C; after: 27.2 ± 0.6°C). Perceived stress level was comparable between Trial 1 (2.4 ± 0.2), Trial 2 (2.3 ± 0.2), and Trial 3 (2.6 ± 0.2). The data did not demonstrate any sex differences for the variables.

**Table 1:** Hemodynamic and thermal response to mental arithmetic

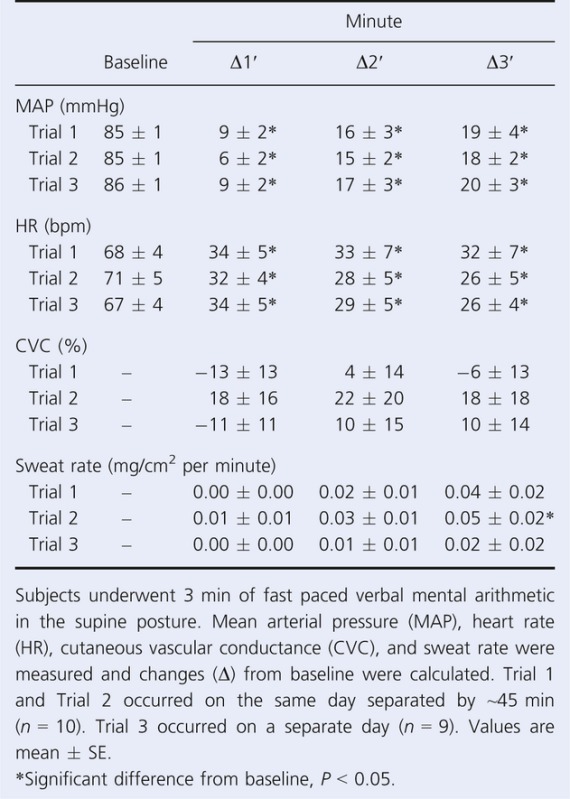

**Figure 2:**
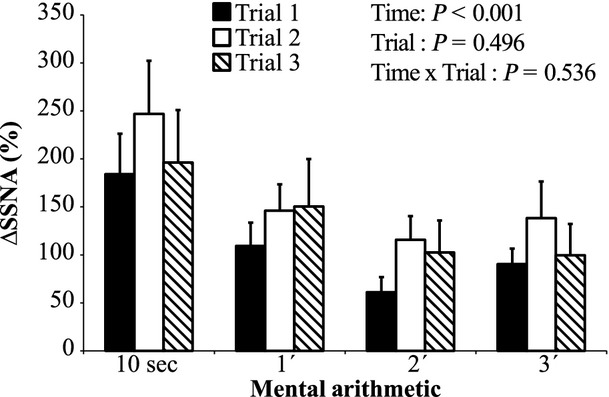
Percent change of skin sympathetic nerve activity (SSNA) during fast paced verbal mental arithmetic (mental stress). Values are mean ± SE, *n* = 9. All values are significantly different from baseline (*P* < 0.001), but the magnitude of increase was not different between trials.

There was moderate test–retest reliability (Cronbach's α = 0.625, *P* = 0.002) in the responsiveness of SSNA to MS between Trial 1 and Trial 2 (i.e., within the same day, when the electrode was in the same location). Thus, individuals with high SSNA responsiveness during Trial 1 tended to have large responsiveness during Trial 2 but intersubject variability existed. The pattern of SSNA responsiveness was comparable during Trial 3 (i.e., between days when the electrode position was not in the exact location, Fig. 2). Test–retest comparisons for ΔMAP (Cronbach's α = 0.855, *P* = 0.001) and ΔHR (Cronbach's α = 0.890, *P* = 0.001) within the same day were strong.

## Discussion

The purpose of this study was to quantify the effect of MS on SSNA and to determine whether the pattern of SSNA responses was comparable between trials. In support of our hypothesis, SSNA increases were largest at stimulus onset (i.e., the first 10 sec of MS) and were significantly elevated above baseline for the entire 3 min. This pattern of SSNA responsiveness to MS was consistent between trials and test–retest reliability was moderate within the same day. End organ responses demonstrated high variability within and between subjects. Taken together, these data extend previous published reports relating to sympathetic neural control during MS (Iwase et al. 1997; Yamamoto et al. 1997; Carter et al. 2005a; Carter and Ray 2009).

### Physiological responses in the current study

The initial microneurography experiments performed in Sweden ∼40 years ago consistently demonstrated that SSNA increased in response to loud noises, stressful conversation, and mental calculations (Delius et al. 1972; Hagbarth et al. 1972; Hallin and Torebjork 1974; Normell and Wallin 1974; Oberle et al. 1988). However, only one of these studies (Hallin and Torebjork 1974) provided quantitative data, but the stimuli were not standardized (i.e., of different durations and intensities). More recently, reports measuring SSNA in response to MS focused on patients with hyperhidrosis and Guillain–Barre syndrome (Iwase et al. 1997; Yamamoto et al. 1997). Considering this background, we designed and conducted this study using healthy human subjects. Our “qualitative” data (Fig. 1) are consistent with past experiments (Delius et al. 1972; Normell and Wallin 1974), but we expanded the field by also providing “quantitative” results (Fig. 2). Furthermore, we demonstrate that the pattern of SSNA responses to MS is comparable between trials with a large initial “novelty” or “arousal” effect followed by a sustained increase in SSNA relative to baseline. Ito et al. (1996) demonstrated that within 1-sec of exposure to a unique auditory tone, SSNA approximately doubled. Our results support these findings. Taken together, these results suggest that SSNA plays a role in the acute adjustments to arousal stimuli and is also significantly elevated above baseline for the duration of tasks involving rapid mental arithmetic and verbal responses.

The test–retest reliability responses were moderate, but nonetheless further emphasize that MS is a robust stimulus to increase SSNA. Previous studies from our laboratory (Ray and Wilson 2004; Wilson et al. 2006) and others (Vissing et al. 1991; Toma et al. 2011) have demonstrated that SSNA increases 20–500% in response to bouts of exercise, indicating that reflex responses to this type of stimulus are quite variable. Regarding MSNA, resting nerve activity is highly reproducible (Sundlof and Wallin 1977), but to our knowledge this study is the first to provide reproducibility data for SSNA. Before conducting this study, it was not certain if the pattern of SSNA responses to mental arithmetic would be attenuated within a given trial or following repeated bouts (i.e., associated with a learning or habituation effect). To this end, our data clearly indicate the following: (1) SSNA responses to mental arithmetic were stable during the entire 3 min within the same trial; and (2) SSNA responses to mental arithmetic were not attenuated in Trials 2 and 3 compared with Trial 1.

The current HR and MAP data are consistent with previous experiments that quantified acute hemodynamic adjustments to MS (Carter et al. 2008; Yang et al. 2013). Specifically, HR typically increases 15–35 bpm in response to mental arithmetic in the supine posture (Carter and Lawrence 2007; Kuipers et al. 2008; Carter and Ray 2009; Klein et al. 2010; Ray and Carter 2010). In these cited studies, MAP increased by 15–20 mmHg which is comparable to the current data.

In the present experiments, we measured end organ responses within the innervation area of the peroneal nerve (i.e., the dorsal foot) and found that skin temperature and skin blood flow did not significantly change relative to baseline in any trial. Sweat rate demonstrated a small increase in Trial 2 only. It is possible that a longer duration of MS or a different site of measurement (e.g., hand vs. dorsal foot vs. dorsal forearm) may have a different effect on skin blood flow and local sweat rate (Machado-Moreira and Taylor 2012; Machado-Moreira et al. 2012). Our findings coupled with recent publications using emotionally charged images (Brown et al. 2012; Henderson et al. 2012) suggest that direct recordings of SSNA are more robust measures of sympathetic outflow than indirect measures of end organ responses. Future studies are needed to clarify how SSNA responses to MS are altered when the body is either hyperthermic or hypothermic.

### Potential clinical relevance

Resting levels of MSNA are reproducible within the same person over months to years; resting levels of MSNA are also elevated in patients with hypertension, heart failure, obstructive sleep apnea, and the metabolic syndrome (Sundlof and Wallin 1977; Somers et al. 1995; Grassi et al. 1998a). Regarding SSNA, attempts have been made to quantify SSNA in several patient groups and most (Middlekauff et al. 1994; Grassi et al. 1998b; Silber et al. 1998; Park et al. 2008) but not all (Iwase et al. 1997; Yamamoto et al. 1997) studies concluded that resting SSNA (measured as bursts per minute) is similar between patients and healthy control subjects. However, there are several technical issues that may weaken these earlier findings. First, SSNA recordings consist of vasoconstrictor, sudomotor, piloerector, and active vasodilator fibers; determining the precise type of fiber is not technically possible in humans. Second, expressing SSNA as bursts per minute does not account for bursts that have an irregular shape, multiple peaks per burst, or the amplitude of each burst. Characterizing the size or number of SSNA bursts also depends on the location of the recording electrode within the nerve fiber. Third, it is not currently known if resting levels of SSNA predict future health outcomes. This is in contrast to resting levels of MSNA, which correlate with disease severity (Leimbach et al. 1986; Grassi et al. 1998a, 2009) and prognosis (Barretto et al. 2009; Ciarka et al. 2010). Providing a stressor to these patients may be needed to unmask disease-related differences in SSNA responsiveness. For the reasons listed above, comparing resting SSNA between groups has been recently criticized (Young et al. 2009).

We and others believe that reflex SSNA responses to physiological stress are more valuable than measurements obtained at rest (Vissing et al. 1991; Ray and Wilson 2004; Wilson et al. 2004; Young et al. 2009). Fundamentally, sympathetic activation serves to prepare a person for “fight or flight.” Earlier studies with human patients did not perturb the sympathetic nervous system and may have missed the opportunity to detect group differences in SSNA (Middlekauff et al. 1994; Grassi et al. 1998b; Park et al. 2008). In other words, SSNA levels should be low when lying quietly in a thermoneutral room because the triggers of SSNA (thermal and arousal stimuli) are low or absent. This is in contrast to MSNA, which is elevated at rest in patients with cardiovascular disease due to the involvement of MSNA in the tonically active baroreflex, which is clearly impaired in several disease states (Bristow et al. 1969; Carlson et al. 1996; Monahan 2007). In support of our speculation, group differences in reflex SSNA responses to physiological stress have been noted (Silber et al. 1998; Watanabe et al. 2004). Whether the alterations in SSNA are a cause or consequence of disease is not known. Taken together, our current data indicate that mental arithmetic (i.e., a relatively short duration autonomic stressor) elicits large and reproducible increases in SSNA. The current findings could be valuable in future studies evaluating the effectiveness of interventions (e.g., exercise training, pharmacological therapy) on sympathetic outflow.

## Conclusions

Psychological stress is a trigger for adverse cardiovascular events (Dimsdale 2008) and greater responses to laboratory stress have been linked with poor future health outcomes (Light et al. 1992; Chida and Steptoe 2010). The current SSNA data are in response to MS address novel and important integrative physiology concepts that may be useful for future clinical investigators. Specifically, we demonstrate that patterns of SSNA responses to standardized bouts of mental arithmetic are consistent across trials with a large initial arousal response followed by a smaller yet sustained SSNA increase for the remainder of the trial. These results indicate that SSNA responses to MS are reproducible in controlled conditions and that changes observed over time would reflect modification of autonomic regulation. Future studies utilizing pharmacological or exercise therapies in patient populations may further clarify how the human body responds to psychological stress.
